# Missing Link?: Alachlor and Semen Quality

**DOI:** 10.1289/ehp.113-a652a

**Published:** 2005-10

**Authors:** David Gustafson

**Affiliations:** Monsanto Company, St. Louis, Missouri, E-mail: david.i.gustafson@monsanto.com

The article by Swan et al. (2003) suggested that alachlor exposure was linked to reduced sperm quality in fertile men; after publication of the article, Monsanto (St. Louis, MO) began a detailed examination of the issue because the findings were entirely unexpected and inconsistent with both our information and the extensive published literature on alachlor. Most surprisingly, alachlor mercapturate (AM) was reportedly found in the urine of 92% of study participants in Columbia, Missouri. Because this metabolite arises exclusively from exposure to the parent compound, few, if any, detects would be expected based on declining alachlor use (National Agricultural Statistics Service 2005) and our detailed understanding of its biological and environmental properties (Feng et al. 1994). Also, extensive water monitoring studies submitted to the U.S. Environmental Protection Agency (EPA) have shown that parent alachlor occurs very infrequently in both potable wells (Holden et al. 1992) and drinking water from surface water sources (Hackett et al. 2005), thereby calling into question the plausibility of such widespread exposure.

We met with Swan and colleagues at the University of Missouri in Columbia (UMC), and with the personnel at the Centers for Disease Control and Prevention (CDC) who conducted the analyses, to discuss our surprise at the findings. Our concern about the reported frequent detections of alachlor in urine was heightened when we learned that the liquid chromatography/mass spectrometry–mass spectrometry method employed by the CDC (Olsson et al. 2004) included no confirmatory ions, a standard technique for avoiding false positives in the analysis of urine (Department of Health and Human Services 1998). An 18-month collaboration ensued, which included numerous discussions and a round-robin study conducted between Monsanto and the CDC, with involvement by UMC researchers, to assess the performance and transferability of the methods used by each laboratory.

After successful completion of the round-robin study, several frozen urine samples retained from the original study were sent to Monsanto. In results that we intend to submit for publication, we found no detectable level of AM (limit of detection < 0.10 ppb) in samples that the CDC reported to contain up to 3 ppb (Swan et al. 2003). Sample degradation does not explain this difference, because we have data demonstrating AM stability under such conditions. Our analyses followed Good Laboratory Practice standards [U.S. Environmental Protection Agency (EPA) 1989] and included confirmatory ions. We also analyzed urine samples of 52 volunteers from agricultural areas across North America, none of which actually contained detectable AM, but 11 of which showed false positives when confirmatory ions were not used.

The CDC has now modified its method to include confirmatory ions for alachlor. We are confident that little or no AM would have been detected had they included confirmatory ions in the original analysis. The CDC previously declined Monsanto's request that they analyze the original samples using a modified method with confirmatory ions. However, after receiving an earlier version of this letter, the CDC quickly performed new analyses of 14 retained frozen samples and informed us that analysis with confirmatory ions validated their original findings. Unfortunately, the CDC has not provided us with sufficient data to confirm the validity of the new method and results. Monsanto continues to believe the detections are spurious. From our perspective, the only possible resolution of the matter at this time would be for the retained samples to be sent to an independent third-party laboratory for confirmatory analysis.

We understand Swan et al. are now having urine samples from other similar agricultural areas analyzed using a confirmatory method, and that AM is no longer being routinely detected. This would affirm that alachlor exposure is rare and the alleged link to semen quality is implausible. It would be very informative to identify the apparent interferent using high-resolution mass spectrometry methodology, but our collaboration has clearly shown that it was not AM. The evidence presented by our analyses of the samples provided by Swan, supported by our successful performance in the round-robin analysis of fortified samples, demonstrates that the reported detections of alachlor were most likely attributable to an interferent. The data refute any link between alachlor exposure and reduced sperm quality in fertile men.
